# Hydrogen Sulfide Prevents Advanced Glycation End-Products Induced Activation of the Epithelial Sodium Channel

**DOI:** 10.1155/2015/976848

**Published:** 2015-05-11

**Authors:** Qiushi Wang, Binlin Song, Shuai Jiang, Chen Liang, Xiao Chen, Jing Shi, Xinyuan Li, Yingying Sun, Mingming Wu, Dan Zhao, Zhi-Ren Zhang, He-Ping Ma

**Affiliations:** ^1^Departments of Clinical Pharmacy and Cardiology, Institute of Clinical Pharmacy, The Second Affiliated Hospital, Harbin Medical University, Key Laboratories of Education Ministry for Myocardial Ischemia Mechanism and Treatment, Harbin 150086, China; ^2^Department of Physiology, Emory University School of Medicine, Atlanta, GA 30322, USA

## Abstract

Advanced glycation end-products (AGEs) are complex and heterogeneous compounds implicated in diabetes. Sodium reabsorption through the epithelial sodium channel (ENaC) at the distal nephron plays an important role in diabetic hypertension. Here, we report that H_2_S antagonizes AGEs-induced ENaC activation in A6 cells. ENaC open probability (*P*_*O*_) in A6 cells was significantly increased by exogenous AGEs and that this AGEs-induced ENaC activity was abolished by NaHS (a donor of H_2_S) and TEMPOL. Incubating A6 cells with the catalase inhibitor 3-aminotriazole (3-AT) mimicked the effects of AGEs on ENaC activity, but did not induce any additive effect. We found that the expression levels of catalase were significantly reduced by AGEs and both AGEs and 3-AT facilitated ROS uptake in A6 cells, which were significantly inhibited by NaHS. The specific PTEN and PI3K inhibitors, BPV_(pic)_ and LY294002, influence ENaC activity in AGEs-pretreated A6 cells. Moreover, after removal of AGEs from AGEs-pretreated A6 cells for 72 hours, ENaC *P*_*O*_ remained at a high level, suggesting that an AGEs-related “metabolic memory” may be involved in sodium homeostasis. Our data, for the first time, show that H_2_S prevents AGEs-induced ENaC activation by targeting the ROS/PI3K/PTEN pathway.

## 1. Introduction

Advanced glycation end-products (AGEs) are modified proteins or lipids that become nonenzymatically glycated and oxidized after contacting aldose sugars and polypeptides. High levels of glucose react with proteins to form adduct AGEs in diabetes mellitus [1]. Increasing evidence suggests that AGEs play an important role in the development of diabetic nephropathy. Typically, proteins after being directly cross-linked to AGEs can change cellular structure and function or may interact with a combination of different cell surface receptors [1, 2]. The long-term progression of diabetic complications in kidney could be a metabolic memory phenomenon. In other words, even after hyperglycemia is efficiently controlled previous exposure of the target cells to high glucose (HG) may still cause the persistence of its deleterious effects. Hypertension is a major complication in diabetes and is the cause of the increasing morbidity and mortality in diabetic patients. Hypertension alone accounts for nearly 85% of cardiovascular disease (CVD) risk factors. Since diabetic patients tend to have higher blood pressure than nondiabetic patients [3], after becoming hypertensive they should have even higher risk for CVD. Therefore, control of the development of hypertension in diabetic patients is very critical for preventing CVD. Recent studies have demonstrated that AGEs are upregulated in hypertensive diabetic subjects, particularly in distal nephron cells [4]. AGE accumulation mediates proliferation, migration, metabolic memory, and inflammatory gene expression in the distal nephron, which is thought to accelerate hypertension development in diabetes [5]. However, the detailed mechanisms underlying hypertension in diabetic patients are not fully understood.

The epithelial sodium channel (ENaC) mediates Na^+^ absorption across epithelial cells in the kidney collecting duct, lung, distal colon, and sweat duct. Na^+^ transport is critical for Na^+^ homeostasis and thus plays a vital role in maintaining salt balance and systemic blood pressure. ENaC excess activation causes hypertension, as seen in Liddle's syndrome [6]. In type 1 and type 2 diabetic animal models, the expression levels of ENaC were increased in cortical collecting duct cells. Cultured with AGEs, ENaC was increased at both mRNA and protein levels in mouse CCD cells [7]. Therefore, it is very possible that AGEs may be involved in the development of hypertension in diabetes, at least, in part, by stimulating ENaC function.

Hydrogen sulfide (H_2_S) is an important intercellular gaseous messenger molecule that regulates multiple physiological and pathological processes. Accumulating evidence has shown that H_2_S protects against a number of organ injuries. One of the primary mechanisms of H_2_S protection is antioxidation, as it either enhances reduced glutathione (GSH, a major cellular antioxidant) [8] or directly scavenges superoxide [9], H_2_O_2_ [10], and peroxynitrite [11] to suppress oxidative stress. Our previous studies suggest that H_2_S could protect H_2_O_2_-induced ENaC activity in A6 cells [12]. Therefore, we hypothesized that AGEs might elevate ENaC activity and that H_2_S might protect against this elevation. The present study shows that H_2_S prevents AGEs-induced ENaC activation by targeting the ROS/PI3K/PTEN pathway.

## 2. Materials and Methods

### 2.1. Cell Culture

A6 cells are an established renal cell line derived from the* Xenopus laevis *distal nephron segment, which is an appropriate cell model for studying ENaC [12]. A6 cells were purchased from American Type Culture Collection (Rockville, MD, USA) and grown in the medium consisting of 3 parts of DMEM/F-12 (1 : 1) medium (Gibco, USA), 1 part of H_2_O, 15 mM NaHCO_3_ (total Na^+^ = 101 mM), 2 mM L-glutamine, 10% fetal bovine serum (Invitrogen, USA), 25 units/mL penicillin, and 25 units/mL streptomycin, as previously described [13]. A6 cells were cultured in plastic flasks in the presence of 1 *μ*M aldosterone at 26°C and 4% CO_2_. After the cells reached 70% confluence, they were subcultured on polyester membranes of* Transwell* inserts (Corning Costar Co, USA) for confocal microscopy or* Snapwell* inserts (Corning Costar Co, USA) for cell-attached patch-clamp analysis. To allow for polarization, cells were cultured for at least 2 to 3 weeks before performing experiments.

### 2.2. Patch-Clamp Recording

ENaC single-channel currents were recorded using a cell-attached patch-clamp configuration with an Axopatch-200B amplifier (Axon Instruments, USA) as described previously [14, 15]. A6 cells were thoroughly washed with NaCl solution containing (in mM) 100 NaCl, 3.4 KCl, 1 CaCl_2_, 1 MgCl_2_, and 10 HEPES, adjusted to pH 7.4 with NaOH. This NaCl solution was used as bath solution for recordings and used to fill the electrodes. The reagents were added to the bath solution from either basolateral side or apical side. Borosilicate glass electrodes had tip resistance of 7–10 MΩ when filled with NaCl solution. Experiments were conducted at room temperature (22–25°C). The data were acquired by application of 0 mV pipette potential and were sampled at 5 kHz and low-pass filtered at 1 kHz with Clampex 10.2 software (Molecular Devices, Sunnyvale, CA, USA). Prior to analysis, the single-channel traces were further filtered at 30 Hz. The total number of functional channels in the patch was determined by observing the number of peaks detected on the current amplitude histograms during at least 10 min recording period. The open probability (*P*_*O*_) of ENaC before and after chemical application was calculated with Clampfit 10.2 (Molecular Devices, Sunnyvale, CA, USA). Control ENaC activity was recorded for 2 min after forming the cell-attached mode and ENaC activity stabilized. A single patch was typically recorded for at least 30 min before any experimental manipulation.

### 2.3. Confocal Laser Scanning Microscopy Analysis

Studies were performed using confocal microscopy (Olympus Fluoview 1000, Japan) as previously described [12, 13, 16]. A6 cells were washed twice with NaCl solution prior to the performance of any experiments. Immediately following experimental manipulation, the polyester membrane support was quickly excised and mounted on a glass slide with a drop of NaCl solution to keep the cells alive. A6 cells grown on Transwell inserts were loaded with 2.5 *μ*M 5-(and-6)-carboxy-2′,7′-dichlorodihydrofluorescein diacetate (carboxy-H_2_DCFDA), a membrane-permeable ROS-sensitive fluorescent probe (Invitrogen, USA) that becomes fluorescent when oxidized. Prior to exogenous AGEs or 3-AT application, A6 cells were treated with an iron chelator, 50 *μ*M 2,2′-dipyridyl for 3 min [17]. Labeled cells were washed twice in modified DPBS before confocal microscopy analysis. ROS levels were measured by fluorescence intensity.

### 2.4. Western Blotting

A6 cells were cultured as described above. Cell lysates (100 *μ*g) were loaded and electrophoresed on 10% SDS-polyacrylamide gels for 60–90 min. Gels were blotted onto polyvinylidene fluoride (PVDF) membranes for 1.5 h at 200 mA. After 1 h of blocking in 5% nonfat dry milk in phosphate-buffered saline (PBS), PVDF membranes were incubated with a rabbit polyclonal primary antibody (1 : 2,000) against catalase (Abcam, ab16731) overnight at 4°C and then incubated with a horseradish peroxidase-conjugated secondary antibody (Santa Cruz Biotechnology, USA, 1 : 5000) for 1 h at room temperature after four vigorous washes. Finally, blots were visualized by chemiluminescence using the ECL Plus Western blotting detection system (Bio-Rad, USA).

### 2.5. Chemicals and Reagents

Unless otherwise noted, all chemicals and reagents were purchased from Sigma Aldrich (St. Louis, MO, USA). AGEs were purchased from Cell Biolabs (San Diego, USA). All solutions were premade and stored in a −20°C freezer or freshly made before use.

### 2.6. Data Analysis

Data are presented as mean ± S.E. Statistical analysis was performed with SigmaPlot and SigmaStat software (Jandel Scientific, CA, USA). Student's* t*-test was used to compare pre- and posttreatment activities. Analysis of variance (ANOVA) was used to make multiple comparisons among various treatment groups. Differences were considered statistically significant when *P* < 0.05.

## 3. Results

### 3.1. H_2_S Reverses AGEs-Induced ENaC Activity in A6 Cells

**Figure 1:**
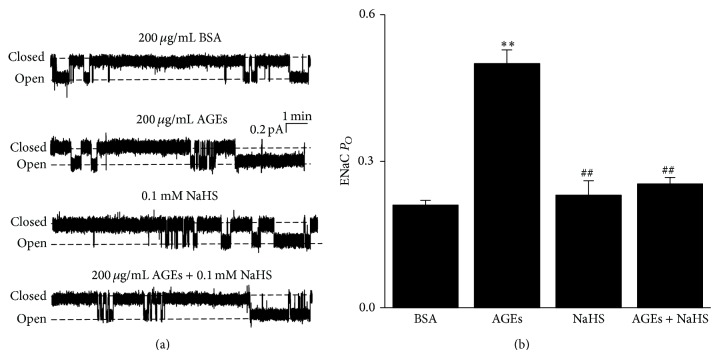
AGEs-induced activation of ENaC is reversed by 0.1 mM NaHS in A6 cells. (a) The representative ENaC single-channel current recorded from A6 cells, respectively, treated with basolateral 200 *μ*g/mL BSA (control; top trace), basolateral 200 *μ*g/mL AGEs, apical 0.1 mM NaHS, and basolateral 200 *μ*g/mL AGEs + apical 0.1 mM NaHS (bottom trace) for 24 h. (b) Summary plot shows that AGEs treatment significantly increased ENaC *P*_*O*_, which was reversed by H_2_S treatment (*n* = 10 for each individual experimental set; ∗∗ indicates *P* < 0.01 compared to control; ## indicates *P* < 0.01 compared to AGEs treated cells).

To investigate whether AGEs enhance ENaC activity, we performed cell-attached patch-clamp experiments. Because in diabetic patients AGEs are delivered to renal epithelial cells from the blood, we applied AGEs to the basolateral side of A6 cell monolayer to mimic the* in vivo* AGEs delivery. We compared ENaC *P*_*O*_ in cell-attached patches from four experimental groups: control (basolateral incubation of A6 cells with 200 *μ*g/mL BSA for 24 h), AGEs (cells treated with basolateral 200 *μ*g/mL AGEs for 24 h), NaHS (cells treated with 0.1 mM NaHS for 30 min; in addition, NaHS at 0.05, 0.1, or 0.3 mM does not affect cell viability [12]), and AGEs + NaHS (cells treated with basolateral 200 *μ*g/mL AGEs for 24 h and then incubation with 0.1 mM NaHS 30 min). After treatment with AGEs, ENaC *P*_*O*_ was significantly increased, from 0.22 ± 0.03 (control; *n* = 10) to 0.50 ± 0.03 (AGEs; *n* = 10; *P* < 0.01 compared to control). Consistent with our previous studies [12], NaHS did not affect ENaC *P*_*O*_ compared with control (0.23 ± 0.03; *n* = 10; *P* > 0.05). The AGEs-induced increase in ENaC *P*_*O*_ was reversed by NaHS (0.25 ± 0.01; *n* = 10; *P* < 0.01 compared to AGEs and *P* > 0.05 compared to control) (Figure 1). These results suggest that AGEs strongly stimulate ENaC activity in A6 cells and that H_2_S exerts a sufficient protective effect on AGE-induced ENaC activity.

### 3.2. Inhibition of Catalase Activity Mimics the Effect of AGEs on ENaC

**Figure 2:**
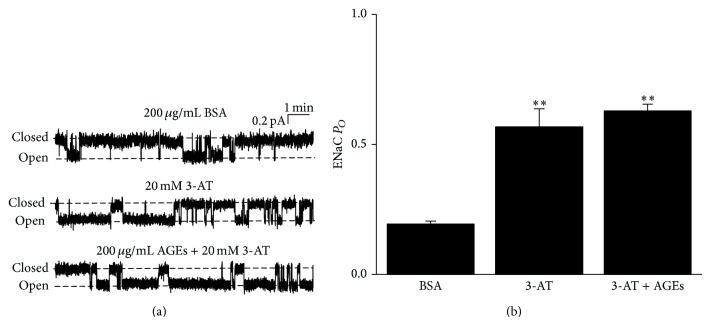
3-Aminotriazole (3-AT) mimics the effect of AGEs on ENaC *P*_*O*_. (a) The representative single-channel currents of ENaC recorded under control conditions (basolateral 200 *μ*g/mL BSA for 24 h; top), after apical 20 mM 3-AT treatment for 30 min (middle), or after 24 h AGE treatment followed by treatment with apical 20 mM 3-AT for 30 min (bottom). (b) Summary plots show that ENaC *P*_*O*_ was significantly, respectively, increased after 20 mM 3-AT treatment (*n* = 10 for control and *n* = 9 for 3-AT group; ∗∗ indicates *P* < 0.01 compared to control). Addition of 3-AT to AGEs did not further increase ENaC *P*_*O*_ compared to 3-AT alone (*n* = 9 for AGEs + 3-AT group; *P* > 0.05).

Because AGEs potently inhibit catalase and ROS regulates ENaC [12, 13, 18], we reasoned that AGEs might stimulate ENaC by increasing ROS levels via inhibition of catalase. Therefore, we used a catalase inhibitor, 3-AT [19], to treat A6 cells. As shown in Figure 2(a), ENaC activity was significantly upregulated by application of 20 mM 3-AT to the basolateral bath; ENaC *P*_*O*_ was increased from 0.22 ± 0.03 (control; *n* = 10) to 0.57 ± 0.07 (*n* = 9; *P* < 0.01 compared to control) (Figure 2(b)). We have to note that 20 mM 3-AT led to an increase in osmolarity from 268 ± 2 mOsmol/kg (*n* = 3) to 302 ± 3 mOsmol/kg (*n* = 3). However, increasing in osmolarity up to 400 mOsmol/kg (adjusted by sucrose) did not affect ENaC *P*_*O*_ in A6 cells (data not shown), which suggests that the effect of 3-AT on ENaC is not due to changes in osmolarity. The combined application of AGEs to the basolateral bath and 3-AT to the apical bath did not further upregulate ENaC (*P*_*O*_ = 0.63 ± 0.03; *n* = 9; *P* > 0.05 compared to 3-AT alone) (Figures 2(a) and 2(b)). These results suggest that AGEs and 3-AT may activate ENaC through the same pathway associated with catalase activity and accumulation of ROS.

### 3.3. H_2_S Attenuates Both AGEs- and 3-AT-Induced Oxidative Stress in A6 Cells

**Figure 3:**
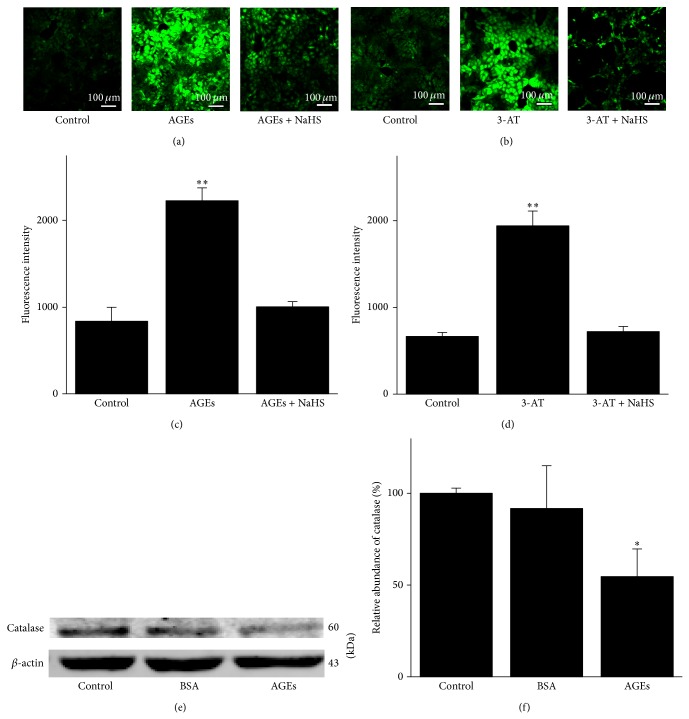
H_2_S ameliorates AGE- or 3-AT-elicited oxidative stress and AGEs reduce catalase expression in A6 cells. (a) The left image shows that there was a residual level of intracellular ROS under control condition; the middle image shows a significant increase in intracellular ROS upon application of basolateral 200 *μ*g/mL AGEs; the right image shows that the AGE-induced increase in intracellular ROS was abolished by 0.1 mM NaHS treatment. (b) The left image shows that there was a residual level of intracellular ROS under control conditions; the middle image shows a significant increase in intracellular ROS upon application of apical 20 mM 3-AT; the right image shows that the 3-AT-induced increase in intracellular ROS was also abolished by 0.1 mM NaHS treatment. (c) and (d) Summarized bar graphs show the mean fluorescence intensities under indicated experimental conditions (*n* = 7 for each experimental condition; ∗∗ indicates *P* < 0.01 compared to control). (e) and (f) Western blot demonstrating that expression levels of catalase were suppressed by AGEs (*n* = 6, ∗ represents *P* < 0.05 compared to control).

AGEs are known to inhibit catalase expression [18]. The inhibition of oxidative stress accounts for the cardioprotective effects of H_2_S during ischemia/reperfusion (I/R) [20, 21]. However, it is unknown whether H_2_S can reduce AGE-induced or 3-AT-induced oxidative stress in A6 cells. Therefore, we examined intracellular ROS levels with an ROS-sensitive fluorescent probe, DCF (refer to Section 2), in the presence of AGEs, AGEs + NaHS, 3-AT, or 3-AT + NaHS. Our results show that pretreatment of A6 cells with AGEs or 3-AT induced significant increase in intracellular fluorescence intensity. These results suggest that exogenous AGEs or 3-AT significantly elevated intracellular ROS in A6 cells. Furthermore, application of 0.1 mM NaHS for 30 min led to a significant decrease in intracellular ROS levels, as suggested by reduced fluorescence intensity upon incubation with NaHS (Figures 3(a)–3(d); *n* = 7). Our Western blotting data show that AGEs caused a significant decrease in catalase expression in A6 cells (Figure 3(e); *n* = 6). These results together suggest that AGEs increase intracellular ROS via inhibition of catalase and H_2_S significantly attenuates AGEs and 3-AT induced ROS accumulation in A6 cells.

### 3.4. TEMPOL Abolishes Both AGEs- and 3-AT-Induced Activation of ENaC

**Figure 4:**
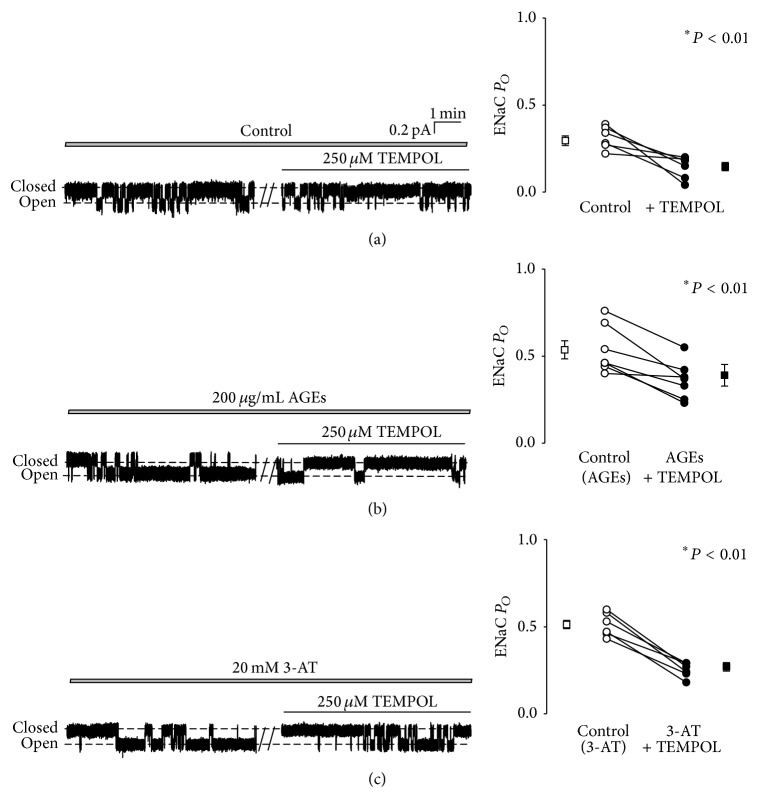
TEMPOL abolishes the effects of AGEs and 3-AT on ENaC activity. (a) ROS extraction by 250 *μ*M TEMPOL significantly decreased ENaC *P*_*O*_ (*n* = 6 paired experiments; ∗ represents *P* < 0.01). (b) and (c) TEMPOL significantly reduced ENaC activity in cells pretreated with 200 *μ*g/mL AGEs (b) or in the cells pretreated with 20 mM 3-AT (c) (*n* = 7 paired experiments; ∗ represents *P* < 0.01).

2,2,6,6-Tetramethyl-1-piperidinyloxy (TEMPOL) is a well-known scavenger used to remove ROS from the cells. To confirm the role of ROS in stimulating ENaC activity, we added 250 *μ*M TEMPOL to the basolateral bath. Our data show that even under control conditions ENaC *P*_*O*_ was significantly decreased by addition of TEMPOL, from 0.30 ± 0.03 (before) to 0.15 ± 0.02 (15 min after TEMPOL) in A6 cells (*n* = 6; *P* < 0.01) (Figure 4(a)). ENaC *P*_*O*_ was also decreased by TEMPOL, from 0.52 ± 0.05 (*n* = 7) to 0.33 ± 0.05 in AGEs treated cells (*n* = 7; *P* < 0.01) (Figure 4(b)) or from 0.51 ± 0.02 (*n* = 7) to 0.27 ± 0.02 in 3-AT treated cells (*n* = 7; *P* < 0.01) (Figure 4(c)). These data suggest that AGEs stimulate ENaC in A6 cells via a pathway closely associated with altered intracellular ROS.

### 3.5. AGEs Elevate ENaC *P*_*O*_ through the PTEN and PI3K Pathways

**Figure 5:**
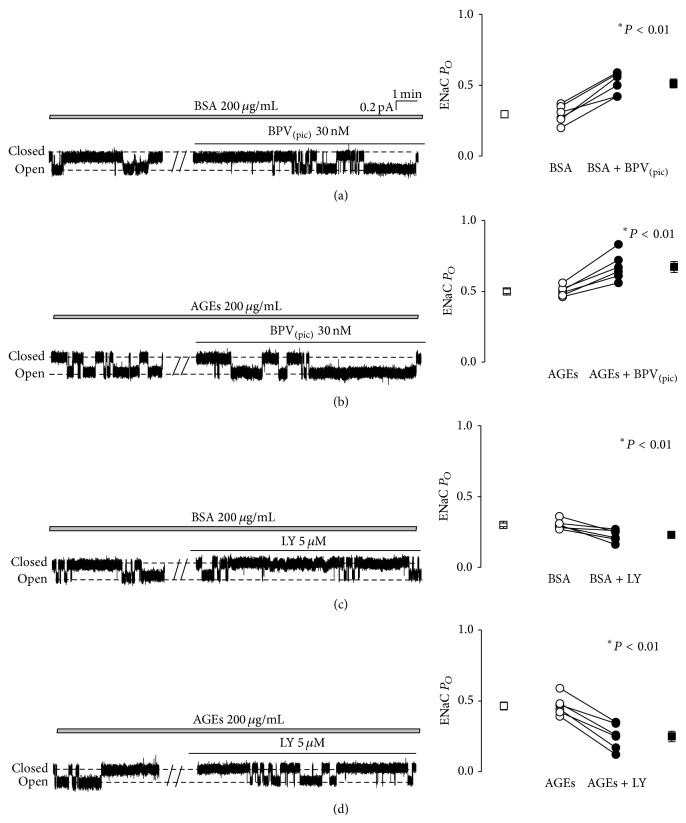
AGEs activate ENaC via PI3K and PTEN signaling pathways. (a) and (b) ENaC activity in A6 cells treated either with basolateral 200 *μ*g/mL BSA or with basolateral 200 *μ*g/mL AGEs, before and after addition of 30 nM BPV_(pic)_ to the apical bath. (c) and (d) ENaC activity in A6 cells treated as in (a) and (b), before and after addition of 5 *μ*M LY294002 to the apical bath; the data show that a PI3K inhibitor, LY294002, significantly inhibits ENaC activities under control condition and in the presence of AGEs. Four breaks between the traces indicate 20 min omitted recording periods. Summarized *P*_*O*_ of ENaC before and after application of each reagent were shown on the right. *n* = 6 paired experiments. ∗ indicates *P* < 0.01.

It is known that the tumor suppressor phosphatase and tension homolog (PTEN) reduces the cellular concentration of PI(3,4,5)P_3_ and acts as a negative regulator of PI3K signaling pathways [22]. Our previous data show that increased intracellular ROS regulates ENaC via increased apical PI(3,4,5)P_3_ by affecting both PTEN and PI3K [12, 13]. Therefore, we tested whether AGEs-induced activation of ENaC is mediated by PTEN or PI3K. Consistent with our previous findings [12], ENaC *P*_*O*_ was increased by ~70% (from 0.30 ± 0.03 to 0.51 ± 0.03; *n* = 6; *P* < 0.05) in A6 cells treated with a specific PTEN inhibitor, BPV_(pic)_ (Figure 5(a)). In AGEs-pretreated cells, ENaC *P*_*O*_ was also significantly elevated by BPV_(pic)_, from 0.50 ± 0.02 to 0.67 ± 0.04 (*n* = 6; *P* < 0.05), but with a less extent (~34%) (Figure 5(b)). LY294002, a PI3K inhibitor, significantly decreased ENaC *P*_*O*_ in untreated control A6 cells (from 0.29 ± 0.02 to 0.23 ± 0.04, reduced ~21%; *n* = 6; *P* < 0.01) (Figure 5(c)). However, in AGEs-pretreated cells, LY294002 reduced ENaC *P*_*O*_ by ~46% (0.46 ± 0.03 versus 0.25 ± 0.04; *n* = 6; *P* < 0.01) (Figure 5(d)). These data together suggest that the inhibition of TPEN and PI3K is involved in AGEs-induced activation of ENaC in A6 cells.

### 3.6. Metabolic Memory Effects of AGEs on ENaC Activity

**Figure 6:**
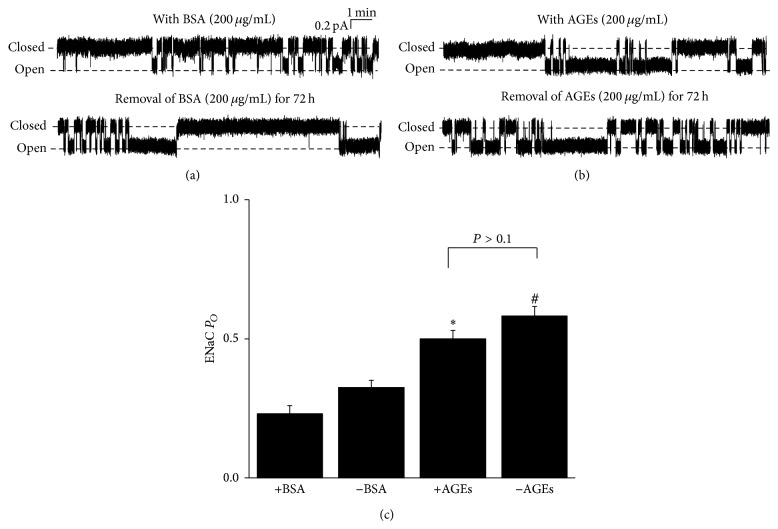
AGEs-induced aberrant activation of ENaC in A6 cells exerts “metabolic memory.” (a) Representative ENaC single-channel currents recorded either from an A6 cell in the presence of basolateral 200 *μ*g/mL BSA or from an A6 cell after removal of 200 *μ*g/mL BSA for 72 h. (b) Representative ENaC single-channel currents recorded either from an A6 cell in the presence of basolateral 200 *μ*g/mL AGEs from an A6 cell after removal of 200 *μ*g/mL AGEs for 72 h. (c) Summarized bar graph shows that basolateral AGEs significantly increased ENaC *P*_*O*_ and the ENaC *P*_*O*_ remained at the same levels after removal of AGEs for 72 h (*n* = 7–10; ∗ and #, resp., indicate *P* < 0.01).

Since AGEs potently upregulate ENaC activity, we hypothesized that AGEs may exert a sustained stimulatory effect on ENaC after withdrawing AGEs, which is called “metabolic memory.” To test this hypothesis, we firstly treated the A6 cells with the medium containing BSA for 24 h and then removed BSA from the medium to continuously culture the cells for 72 h. We also cultured the cells with a medium containing AGEs for 24 h and then removed AGEs from the medium (AGEs-free) followed by continuously culturing these cells for another 72 h. As seen in Figure 6, removal of BSA did not affect ENaC *P*_*O*_ (Figures 6(a) and 6(c)). Consistent with previous results, AGEs significantly increased ENaC *P*_*O*_ (0.50 ± 0.03; *n* = 7); interestingly, ENaC *P*_*O*_ remained at the similar high levels in the cells 72 h after removal of AGEs (*n* = 10; *P* > 0.1) (Figures 6(b) and 6(c)). These results suggest that AGEs regulate ENaC with metabolic memory.

## 4. Discussion

Our major findings in this study are as follows: (1) AGEs stimulate ENaC by elevating intracellular ROS via inhibition of catalase; (2) NaHS reverses the effects of AGEs on ENaC activity by reducing AGEs-induced accumulation of intracellular ROS; (3) AGEs stimulate ENaC with “metabolic memory”; and (4) AGEs strongly activate ENaC through ROS/PTEN/PI3K singling pathways.

AGEs are produced by long-term challenge with high glucose and polypeptides. AGEs are the important pathogenic factors in diabetic nephropathy; however, almost all previous studies have focused on the mechanisms how AGEs take their effects on glomerular and vascular cells [23]. The effects of AGEs on fine sodium absorption in renal tubule cells, particularly in collecting ducts, have rarely been explored. It has previously been reported that circulating AGEs correspond to approximately 50 mg/mL of AGEs in diabetic patients [24]. Because the amount of AGEs decreases in the urine of diabetic patients, therefore we used* Snapwell* insert to culture A6 cells in order to mimic the biological environment. We then applied AGEs to the basolateral membrane, where renal collecting tubules should be exposed to high concentration of AGEs* in vivo*. Because AGEs may be concentrated in renal tissues* in vivo* and the corresponding levels of AGEs* in vitro* have not been conclusively determined, we examined the effects of a variety concentration of AGEs (up to 500 mg/mL) on cellular viability. Our data show that incubation of A6 cells with AGEs (up to 500 *μ*g/mL) for 24 h did not affect cell viability (data not shown). Therefore, the effects of AGEs on ENaC should not be due to nonspecific effects on cell viability because in all the experiments A6 cells were treated with only 200 *μ*g/mL AGEs. Instead, our results show that AGEs stimulate ENaC in A6 cells through catalase inhibition and subsequently an increase in intracellular ROS levels. We propose that catalase is a major player, because 3-AT, a catalase inhibitor, mimics the effects of AGEs on ENaC activity and intracellular ROS levels, albeit 3-AT at a concentration of 20 mM may lead to saturated increase in ENaC activity; this might result in a possibility that there is a catalase-independent effect of AGEs on ENaC activity. However, our data show that AGEs suppress the expression levels of catalase and that in AGEs treated cells 3-AT was no longer able to further activate ENaC. We also propose that ROS plays a critical role in downstream catalase, because, in the presence of ROS scavenger, TEMPOL, AGEs- and 3-AT-induced activation of ENaC were almost completely abolished. Our studies clearly suggest that ROS mediates the AGEs-induced activation of ENaC in A6 cells. Our results are also consistent with previous studies, where the amiloride-sensitive short-circuit currents across A6 cell monolayer were significantly reduced by extraction of intracellular ROS with TEMPOL [25].

It is well documented that oxidative stress is a primary cause of diabetes-induced kidney injury, which may be involved in diabetic hypertension [1, 2]. Recently, we have shown that H_2_O_2_-induced increase in ENaC activity can be reversed by NaHS (a H_2_S donor) in A6 cells [12]. H_2_S is an endogenous gaseous mediator that exerts various physiological and pathophysiological effects* in vivo*, including antioxidative stress and anti-inflammatory response in heart, liver, kidney, and other organs [26–28]. It was reported that NaHS provided cytoprotection in human neuroblastoma cells exposed to D-galactose and that H_2_S may have potential antiaging effects through a reduction of ROS and AGEs formation [29]. As an endogenous signaling molecule, H_2_S can be as high as 0.1 mM in human blood [30] or about 1.6 nmol/mg in intact rat kidneys [31]. Therefore, a final concentration of 0.1 mM NaHS which was used in this study should represent the physiological concentrations, as we reported previously [12]. Our results show that AGEs-induced activation of ENaC and accumulation of intracellular ROS were completely reversed by 0.1 mM NaHS. These results suggest that H_2_S exerts a protective effect against elevation of intracellular ROS and ENaC activity induced by AGEs.

Our previous studies suggest that an increase in intercellular ROS leads to elevation of PI(3,4,5)P_3_ near the apical compartment of A6 cells [12, 13], which is known to stimulate ENaC [32, 33]. Since the levels of PI(3,4,5)P_3_ near the apical compartment of A6 cells are governed by both PTEN and PI3K [12, 13], we examined both PTEN and PI3K inhibitors on ENaC activity in the absence or in the presence of AGEs. Consistent with previous findings [12, 13], it appears that both PTEN and PI3K are involved in the activation of ENaC by AGEs. Interestingly, it has been reported that there are reduced H_2_S levels in diabetic rats and that H_2_S can increase cellular PI(3,4,5)P_3_ levels and can enhance glucose utilization in adipocytes by activating PI3K and inhibiting PTEN [34, 35]. Moreover, we have shown that in distal nephron epithelial cells increased intracellular ROS elevates PI(3,4,5)P_3_ levels near the apical membrane compartment via PTEN/PI3K to stimulate ENaC [12, 13]. However, it would be difficult to determine whether PTEN and PI3K equally contribute to effects of AGEs on ENaC activity.

Finally, this study also provides an evidence for AGEs to stimulate ENaC via a metabolic memory phenomenon which occurs in the long-term progression of diabetic complications in kidney. This phenomenon describes a surprising persistence of the deleterious effects of high glucose even after hyperglycemia has been tightly controlled. This cellular memory phenomenon was revealed by large-scale multicenter clinical trials such as the Diabetes Control and Complications Trial (DCCT) and the Follow-Up Observational Epidemiology of Diabetes [5]. Interestingly, we found that the effect of AGEs on ENaC activity in A6 cells lasted, at least, for 72 h after removal of AGEs, suggesting that there is a metabolic memory phenomenon in Na^+^ homeostasis. This metabolic memory may play an important role in sustaining activation of ENaC, which accounts for the development of hypertension in diabetic patients.

## 5. Conclusion

AGEs significantly stimulate ENaC activity in A6 cells via inhibition of catalase and the effects of AGEs can be reversed by NaHS. Inhibition of catalase activity accounts for both oxidative stress induced by AGEs and elevation of PI(3,4,5)P_3_ near the apical membrane compartment via PTEN/PI3K signaling pathways, thereby regulating ENaC activity. Finally, the effect of AGEs on ENaC activity exerts a metabolic memory phenomenon in A6 cells.
